# A Multidisciplinary Approach in the Management of a Grade 3 Endo-Periodontal Lesion

**DOI:** 10.7759/cureus.85715

**Published:** 2025-06-10

**Authors:** Oulghazi Ichraq, Mariam Mounsif, Maria Fennich, Amal El Yamani

**Affiliations:** 1 Department of Prosthodontics, Mohammed V University, Rabat, MAR; 2 Department of Periodontology, Mohammed V University, Rabat, MAR; 3 Department of Conservative Dentistry and Endodontics, Mohammed V University, Rabat, MAR

**Keywords:** endo-periodontal lesion, grade 3 periodontal lesion, multidisciplinary approach, periodontal therapy, root canal retreatment

## Abstract

Endodontic-periodontal lesions involve a pathological connection between the pulpal and periodontal tissues, often complicating both diagnosis and treatment. Accurate identification and classification are essential to determine an appropriate therapeutic approach. This case report describes the multidisciplinary management of a grade 3 endo-periodontal lesion without root damage. A 43-year-old female presented with pain and swelling in the left mandibular first molar. Clinical and radiographic findings indicated a failed root canal treatment, deep periodontal pockets, and furcation involvement. After emergency drainage of a periodontal abscess, endodontic retreatment was initiated with sodium hypochlorite irrigation and calcium hydroxide medication, followed by canal obturation and coronal restoration. Non-surgical periodontal therapy was also performed. Over a three-year follow-up, clinical and radiographic evaluations confirmed resolution of symptoms and periodontal stability. This case highlights the importance of a sequential, combined approach and emphasizes the need for a proper coronal seal to ensure long-term success in managing complex endo-periodontal lesions.

## Introduction

Endo-periodontal lesions refer to a pathological communication between the pulpal and periodontal tissues. These lesions can be present in either an acute or chronic form [1]. Numerous classifications for endo-periodontal lesions have been introduced, most of which originate from Simon et al.’s (1972) classification, which includes five categories based on the origin of the infection: primary periodontal lesion, primary periodontal lesion with secondary endodontic involvement, primary endodontic lesion, primary endodontic lesion with secondary periodontal involvement, and true combined lesions [2,3]. The most recent classification was proposed in the 2017 World Workshop on the Classification of Periodontal and Peri-Implant Diseases and Conditions [4]. This classification emphasizes a prognosis-oriented approach, replacing the previous system, which was primarily based on lesion origin. Endo-periodontal lesions are classified into two main categories, based on the presence or absence of root damage such as root fracture, root canal perforation, or external root resorption. In the absence of root damage, the endo-periodontal lesions are further subdivided based on the periodontal status of the patient as either occurring in periodontitis or non-periodontitis patients. Each of these categories is subsequently graded from 1 to 3 based on the severity and complexity of the lesion (periodontal pocket extent and location), with grade 3 indicating the presence of deep periodontal pockets in multiple tooth surfaces [1,2]. This new classification enhances clinical decision-making by aligning diagnostic categories with treatment complexity and prognosis.

Endo-periodontal lesions, particularly grade 3 in periodontitis patients, remain among the most challenging conditions to manage in dental practice due to diagnostic uncertainty, the need for interdisciplinary management, and their poor to hopeless prognosis [1].

This case report illustrates the successful multidisciplinary management of a grade 3 endo-periodontal lesion in a periodontitis patient, affecting the left mandibular first molar, with clinical and radiographic follow-up over three years.

## Case presentation

**Figure 1:**
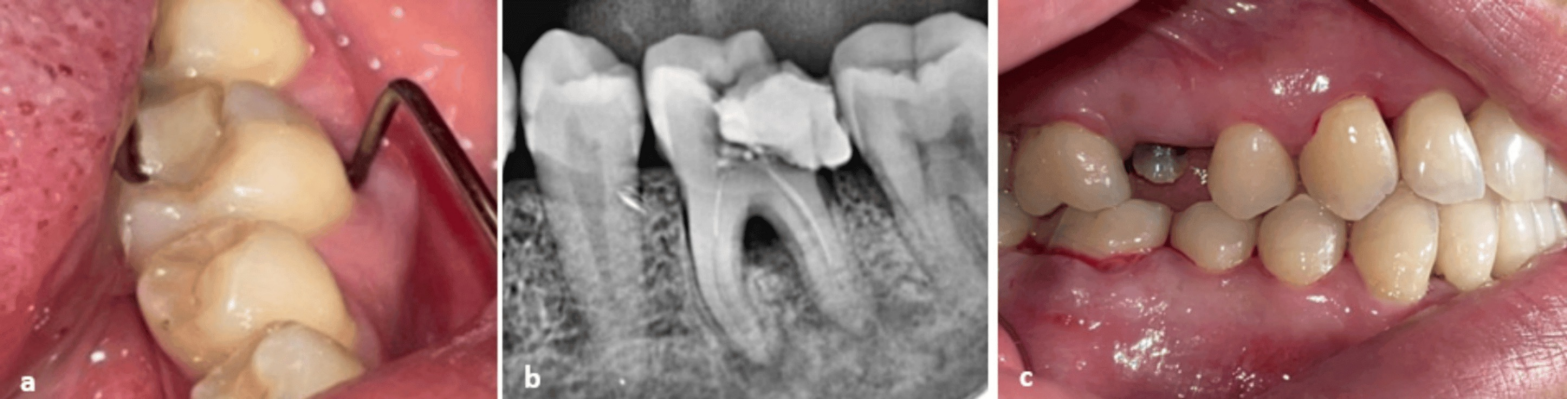
(a) Intraoral view revealing the presence of a periodontal pocket and gingival swelling. (b) Preoperative periapical radiograph showing periapical and interradicular radiolucency. (c) Intraoral view after scaling and drainage of the periodontal abscess.

**Figure 2:**
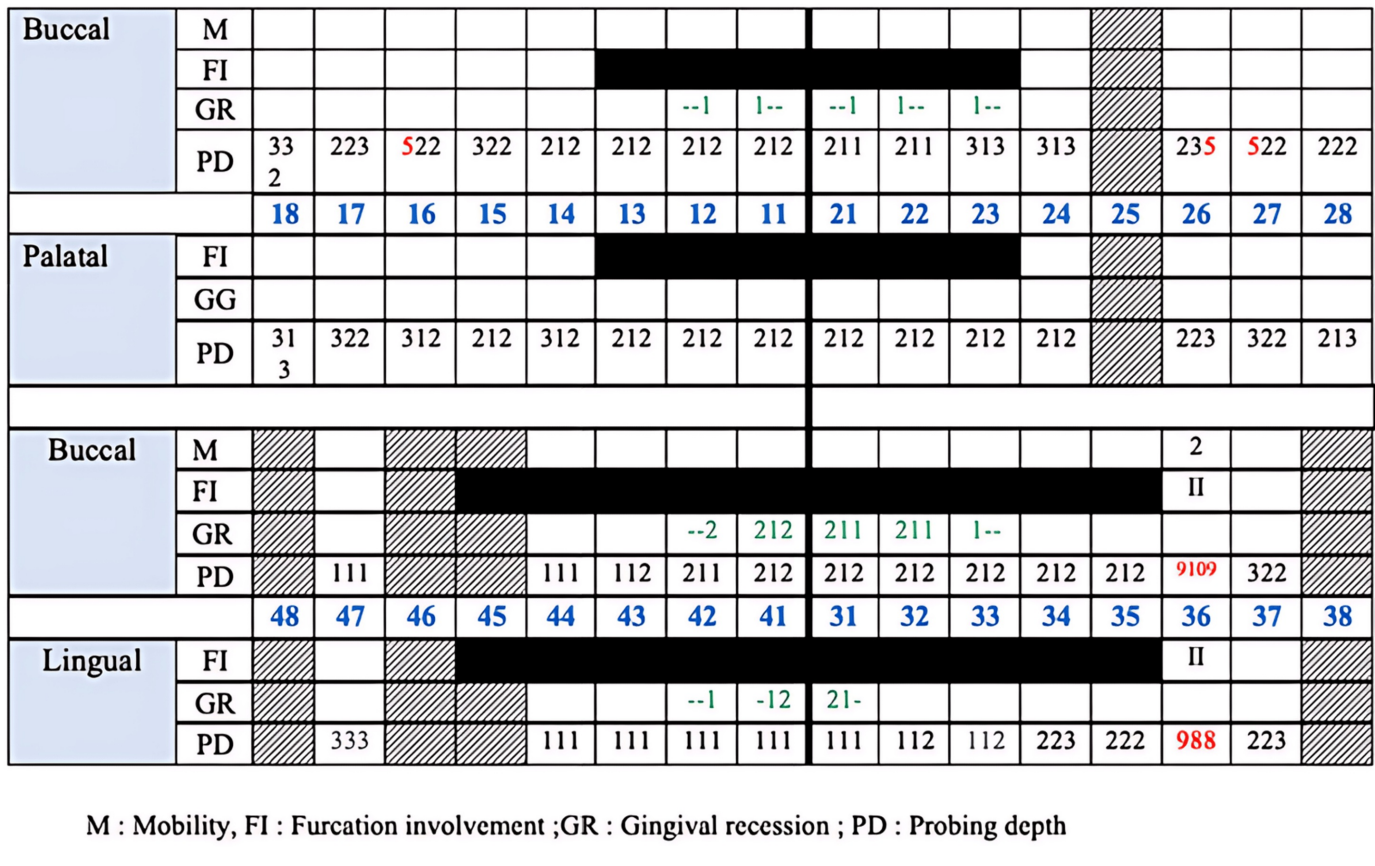
Initial periodontal charting.

A 43-year-old female patient, with a non-contributory medical history, presented to our practice with a chief complaint of swelling and masticatory pain localized to the left mandibular first molar. Intraoral assessment revealed gingival swelling in the buccal side of the affected tooth, a defective coronal composite restoration, and a grade 2 mobility. Periodontal probing pockets measuring 9 mm each were noted (Figures 1A, 2). Periodontal exam revealed a generalized stage II grade B periodontitis, which corresponds to a moderate periodontal destruction with a moderate rate of disease progression, as defined by the 2017 World Workshop classification [4]. Pulp sensitivity testing included a cold test, which showed a negative result. The percussion test was negative. Palpation showed a purulent exudate expressed upon pressure on the buccal side of the affected tooth. Periapical radiography revealed a failed root canal treatment, with periradicular radiolucency affecting both roots and the interradicular region, suggesting furcation involvement, which is the result of the pathologic destruction of periodontal attachment and bone in the interradicular region of multirooted teeth (Figure 1B). Based on the clinical and radiographic findings, a diagnosis of an acute grade 3 endo-periodontal lesion without root damage in a periodontitis patient was established. This corresponds to a true combined endo-periodontal lesion, according to Simon’s classification [3]. An emergency treatment was performed, including drainage of the periodontal abscess at the site of the causal tooth (Figure 1C).

**Figure 3:**
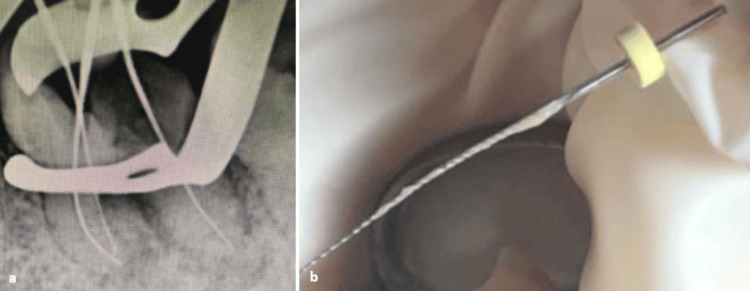
(a) Periapical radiograph with a file in place following canal de-obturation. (b) Endodontic drainage using a file, showing purulent exudate.

Subsequently, endodontic treatment was initiated, starting with the removal of the defective coronal restoration, followed by canal de-obturation (Figure 3A). Endodontic drainage revealed the presence of purulent exudate (Figure 3B).

**Figure 4:**
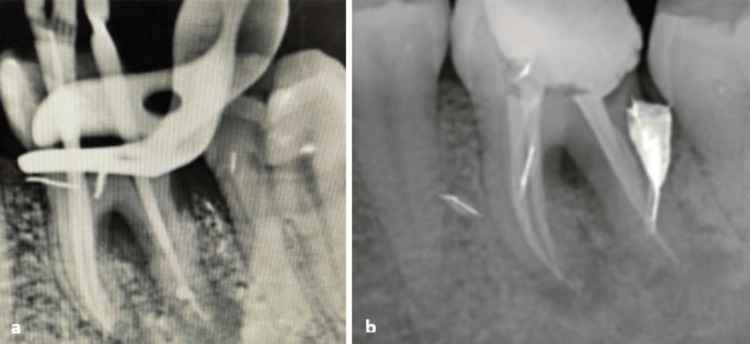
(a) Periapical radiograph with gutta-percha cones. (b) Periapical radiograph after root canal obturation.

**Figure 5:**
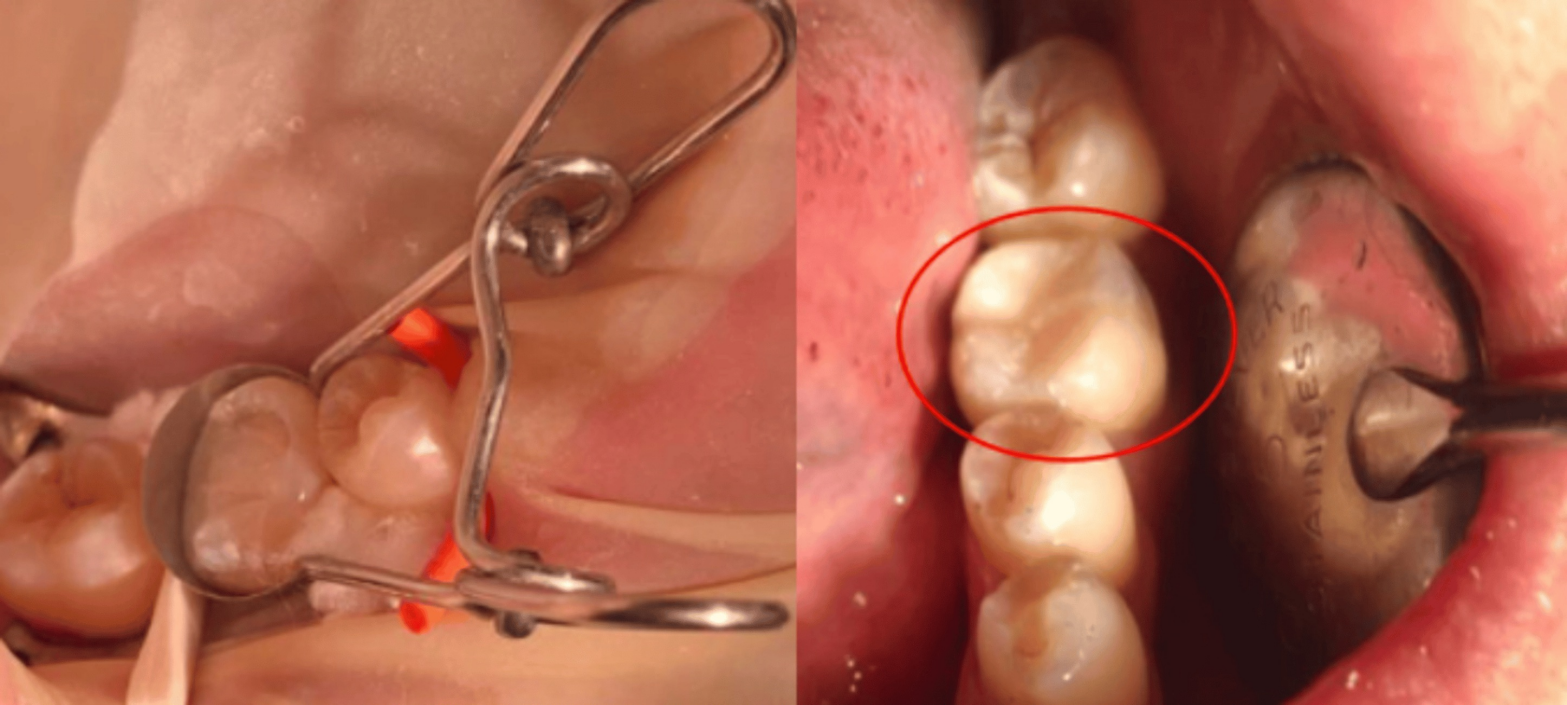
Intraoral view showing coronal restoration with composite resin.

Thorough canal irrigation was performed with 2.5% sodium hypochlorite, followed by the application of intracanal medication with calcium hydroxide. A provisional period of three weeks was observed before completing the root canal obturation (cold lateral condensation technique) (Figure 4) and placing a coronal restoration using glass ionomer cement combined with composite resin (Figure 5).

**Figure 6:**
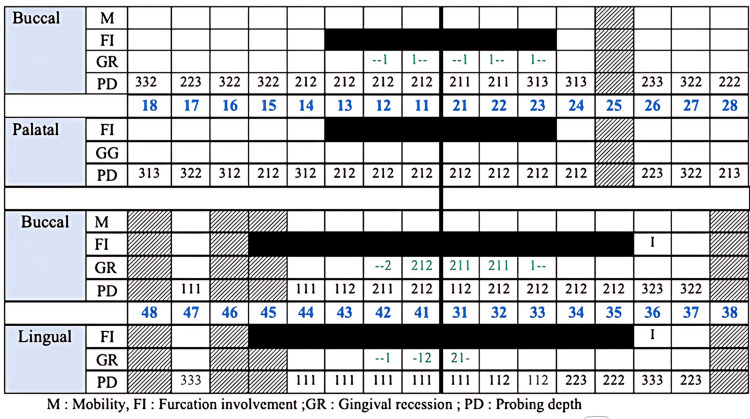
Periodontal charting at three-month postoperative follow‑up.

**Figure 7:**
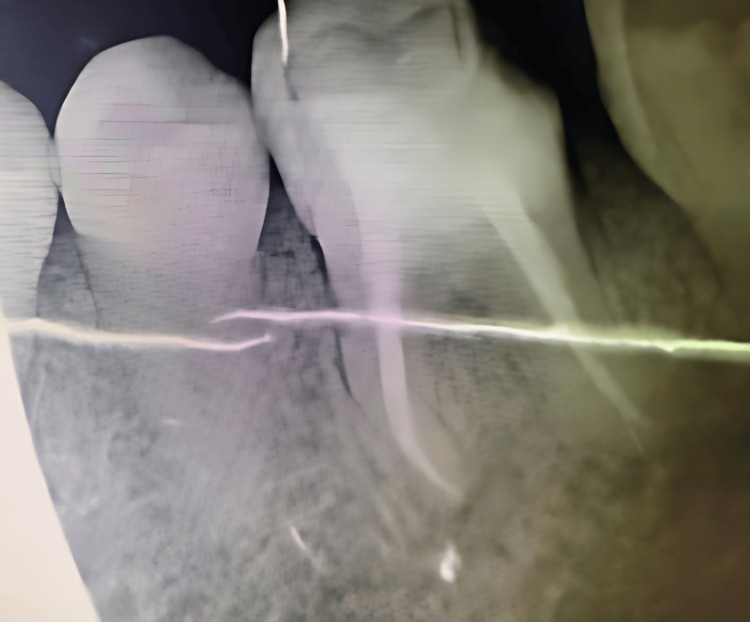
Periapical radiograph demonstrating regression and bone regeneration in the interradicular and periapical regions at one-year follow-up.

Follow-up at three and six months showed positive clinical outcomes: periodontal probing revealed a significant reduction of periodontal pockets (from 9 mm to 3 mm) (Figure 6), and percussion test was negative. At one-year follow-up, the furcation involvement improved from class II to class I on both the buccal and lingual aspects. A radiographic evaluation also demonstrated significant regression of the peri-radicular and interradicular lesions, suggesting bone regeneration (Figure 7). However, distal loss of the coronal restoration was noted (Figure 7).

**Figure 8:**
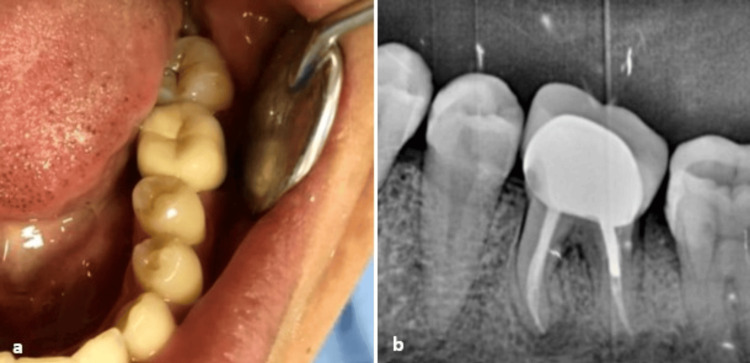
(a) Endobuccal view of the prosthetic restoration. (b) Periapical radiograph showing the prosthetic rehabilitation at three-year follow-up.

To ensure an adequate coronal seal and prevent reinfection, a metal-ceramic crown with a metal post was placed. The successful resolution of periodontal lesion was attributed to the proper management of the corono-endodontic continuum. Three years after the prosthesis cementation, the tooth shows no symptoms and remains periodontally and endodontically stable (Figure 8).

## Discussion

Endo-periodontal lesions present considerable diagnostic, prognostic, and therapeutic challenges, particularly due to the overlapping clinical and radiographic manifestations of periodontal and endodontic pathologies. These challenges are further compounded when pulpal and periodontal involvement occur concurrently. Accurate diagnosis necessitates a comprehensive evaluation of clinical and radiographic parameters, supported by a thorough understanding of the underlying etiology and pathogenesis [5,6]. Our clinical case reports a grade 3 endo-periodontal lesion without root damage in a periodontitis patient.

One of the main advantages of the new classification system of endodontic periodontal lesions is that it also takes into consideration the impact of the signs and symptoms on their prognosis, unlike previous classification systems [4]. The prognosis primarily depends on several factors, including the presence or absence of root damage, the existence of periodontitis, anatomical considerations, and the extent of periodontal destruction affecting the tooth. In our case, we encountered a grade 3 endo-periodontal lesion without root damage in a periodontitis patient, with furcation involvement and no anatomical factors, indicating a poor prognosis [7,8]. Additionally, the greater the portion of the lesion attributed to root canal infection, the more favorable the prognosis for the regeneration of the attachment [9].

Management of endo-periodontal conditions typically requires a multidisciplinary approach. Combined endo-periodontal lesions require completion of endodontic treatment first, followed by periodontal therapy [10]. Clinical evidence supports the use of calcium hydroxide (Ca(OH)_2_) and chlorhexidine as intracanal medicaments, which have been shown to improve periodontal healing outcomes. Periodontal therapy begins with non-surgical interventions, including controlling risk factors such as oral hygiene and systemic conditions, followed by scaling and root planing. If deep periodontal pockets persist despite these treatments, surgical interventions may be required [8,11,12].

However, when the patient presents a periodontal abscess, like in the reported case, it is managed differently. Previous studies suggested that the treatment should be done in two stages, the first consisting of the management of the acute lesion, involving the drainage of the periodontal abscess through the periodontal pocket and subgingival scaling and root planing. The second stage consists of treating the original and residual lesions [5,10].

In our case, the combination of endodontic and non-surgical periodontal treatment resulted in satisfactory results. Permanent restoration must be placed after the completion of endodontic treatment, in order to avoid coronal leakage, considered an important factor influencing tooth survival during and after endodontic-periodontal treatment [13].

Despite the initial diagnosis suggesting a poor prognosis for a grade 3 endo-periodontal lesion in a periodontitis patient, the outcome in this case highlights the potential for long-term stability when a multidisciplinary and effective approach is employed. Early intervention, including prompt drainage of the abscess, sequential endodontic therapy, and subgingival instrumentation, and timely and appropriate restoration of the coronal seal using a definitive prosthesis were key factors contributing to the successful outcome.

## Conclusions

This case highlights the importance of a multidisciplinary approach in the diagnosis and management of endo-periodontal lesions. Careful assessment of clinical and radiographic parameters and appropriate treatment sequencing are essential to achieving favorable outcomes. In this report, the combined endodontic and non-surgical periodontal therapy led to the successful resolution of a grade 3 endo-periodontal lesion without root damage, with clinical stability maintained at three-year follow-up. Timely intervention, effective coronal sealing, and close post-treatment monitoring were key factors contributing to long-term tooth preservation.

However, it is important to recognize that not all grade 3 endo-periodontal lesions, particularly in periodontitis patients, will respond similarly. Prognosis may vary depending on multiple factors, including lesion extent, systemic health, anatomical complexity, and patient compliance.
